# Prenatal Influence?

**Published:** 1916-07

**Authors:** 


### Prenatal Influence?

As to the gross forms of maternal impression, we can say only that every time we satisfactorily adjust our own mind to the logical, scientific, state of scepticism some one publishes a report, perhaps illustrated and at any rate of indubitable authenticity showing, as perfectly as can be shown by the post hoc, propter hoc argument, that maternal impressions do determine foetal development.

The N. Y. Medical Journal has in its issue of Nov. 13, editorially denied not only this crude action of maternal impressions, but the influence of the mental state of the pregnant woman on the offspring. It is held that whatever psychic characteristics are transmitted to offspring, are potentially present in the original reproductive cells. This extension of the logical scepticism as to maternal impressions, does not seem to us wholly logical. The ordinary anatomic and physiologic development of the foetus is not to any large degree a matter of individual heredity but mainly one of racial evolution. That gross departures from the normal development can be caused by transient ocular or psychic impressions seems absurd and, at best, is only occasionally and coincidentally supported by observation. Minor physical characteristics, such as stature, color of hair and eyes and the like, are as strictly material as the general characteristics of two-leggedness, the partition of the extremities into five and the like. But, as has been most thoroughly demonstrated by Mendel and his disciples, even the minor characteristics are by no means determined directly by the parents but represent the same tendencies of racial evolution and distribution as the general ones. The same materialistic argument which denies the possibility of mental—including moral—impressions during pregnancy, might with equal force, be applied to the assumption that the guiding factors in such development are, somehow, included in the reproductive cells.

Under these circumstances, it seems allowable to be guided by empiric observation. We frankly admit that we incline strongly toward the belief that the marked differences noted in the psychic make-up of various children of the same parents, actually reflect the tendencies temporarily present in the parents at the time of conception and, more particularly, those manifested by the mother during the period of pregnancy. That there are numerous exceptions must be admitted, as also that whatever such tendencies may be conceded, may also be overwhelmed by post natal influences. But the tendency to reproduce in successive children the life periods of the mother, seems to us a very general fact. Obviously, mere aspirations on the part of the mother cannot be depended on to produce results beyond the general hereditary possibilities but it does not seem unreasonable to assume that they may have as marked an influence on the development of the psychic characteristics of the child as personal hygiene on the mother's part may have on the physical development of the foetus.

One important practical fact remains. Even one who believes that the mother can in no way influence her child's psychic and mental development by conscious effort, must admit that her aspirations toward high ideals can do no harm and that, in general, they will stimulate her to an observance of hygienic principles which have an admitted influence on the physical and indirectly, the mental and moral development of the child. Whatever danger rests with falsity of opinion, is all one way.

